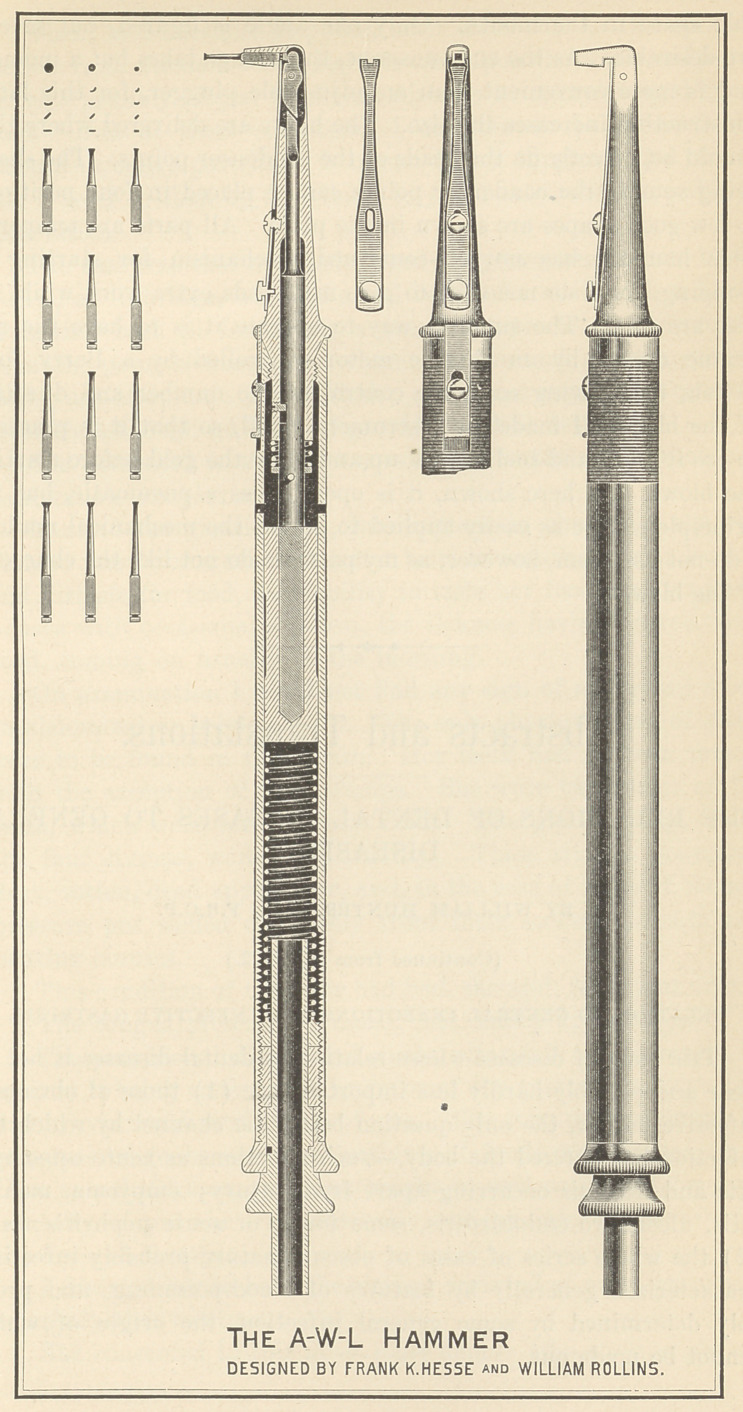# Dental Notes

**Published:** 1899-10

**Authors:** William Rollins


### DENTAL NOTES.

BY WILLIAM ROLLINS.

### NOTE II. A HAMMER.

As the commercial angle mallets are larger and less efficient than is desirable, I illustrate and describe one designed by my friend Mr. Hesse and myself. My aim was to increase the efficiency while reducing the size and number of parts. The drawings have been made with care, of the exact size, to avoid unnecessary description. The instrument is, however, smaller than would appear from the plate. This hammer gives an efficient and comfortable blow, does not obstruct the view of the cavity, and takes little valu-

THE A-W-L HAMMER

DESIGNED BY FRANK K. HESSE AND WILLIAM ROLLINS.

able space in the mouth. Only one angle is figured, but several are desirable. As the ends unscrew, the change takes but a moment and is more convenient than an adjustable plugger, for this latter construction increases the size. The blows are delivered where they should be, directly on the heads of the condenser points. The shafts being square, the condenser points can be placed in four positions. A few good shapes are shown in the plate. All parts are tempered. This hammer has no self-contained mechanism for starting or stopping, for I do not wish to give my hands extra work while my feet are idle. The simplest way to operate it is to have the air-engine driven by an electric motor controlled by a Berry foot-switch, thus giving complete control of the number and duration of the blows. I made the instrument small, so that it is practical to use it as a hand-tool to pick up and place the gold before starting the blows. As here shown, it is operated as a pneumatic, but the principle can be as easily applied to any of the mechanical mallets. I do not use them, however, as my patients do not like the character of the blows.

## Figures and Tables

**Figure f1:**